# Conjunctival Lesions Secondary to Systemic Varicella Zoster Virus Infection

**DOI:** 10.1097/coa.0000000000000022

**Published:** 2023-10-19

**Authors:** Justin J. Grassmeyer, Kellyn N. Bellsmith, Allison R. Bradee, Roma B. Pegany, Travis K. Redd

**Affiliations:** Casey Eye Institute, Oregon Health & Science University, Portland, OR.

**Keywords:** varicella, conjunctivitis, ocular surface disease

## Abstract

**Purpose:**

To report and present images of a case in which discrete conjunctival lesions developed in the setting of primary varicella zoster virus infection (ie, chickenpox).

**Methods:**

Case report and literature review.

**Results:**

This report describes a young, unvaccinated male who developed an acutely painful, red eye in the setting of disseminated primary varicella zoster infection. The cutaneous rash was widespread and included lesions on both eyelids. The patient was found to have multiple discrete de-epithelialized lesions involving the palpebral and bulbar conjunctiva. Throughout the disease course, good visual function was maintained and there was no evidence of intraocular involvement. The ocular surface lesions resolved without sequelae after 1 week of treatment with topical antibiotic ointment.

**Conclusions:**

Primary varicella zoster infection is an increasingly rare phenomenon in the setting of widespread vaccination. However, unvaccinated or undervaccinated individuals and other at-risk populations remain susceptible to developing severe infections. This case of chickenpox involved discrete conjunctival lesions that resolved without sequelae after conservative treatment with topical antibiotic ointment. While serious ophthalmic complications are uncommon in primary varicella infection, clinicians should be aware of the potential for ocular morbidity in this increasingly rare condition.

Varicella zoster virus (VZV) is a highly contagious herpesvirus that spreads primarily via airborne droplets.^1^ It manifests in humans in 2 forms: primary infection, also known as chickenpox; and secondary infection caused by reactivation of latent virus, also known as herpes zoster or shingles. Chickenpox was a nearly universal childhood infection in the United States until a widespread vaccination program was introduced in 1995. Since then, its incidence has decreased by 97%.^1^ Despite the proven efficacy of vaccination, many countries worldwide do not routinely vaccinate children against VZV.^2^

Reactivation along the V1 distribution (ophthalmic branch) of the trigeminal nerve (ie, herpes zoster ophthalmicus) is a common disease with well-known potential for ocular involvement. However, ocular complications from primary disseminated varicella infection are relatively less frequent, with few published descriptions and images of ocular involvement. Among pediatric patients with chickenpox, reported ocular involvement during the acute illness phase includes conjunctivitis,^3–5^ keratitis,^3–6^ uveitis,^3–8^ internal ophthalmoplegia,^6,9–11^ retinitis,^12^ and optic neuritis.^3,12^ The case reported here involves eyelid and ocular surface lesions of the palpebral and bulbar conjunctiva without corneal, uveal, retinal, or optic nerve involvement. This case describes chickenpox infection with ocular involvement, an increasingly rare condition that carries the potential for serious ocular sequelae if unrecognized or untreated.

## CASE REPORT

A 10-year-old male unvaccinated for varicella was brought to the emergency department by his mother for 3 days of worsening right eye redness and irritation that developed concurrently with a widespread pruritic rash. His older brother, also unvaccinated for varicella, had chickenpox 3 weeks prior, which resolved without complication. The patient's rash started on his chest and spread widely across his body, including his trunk, legs, arms, and face, including his eyelids bilaterally. He denied symptomatic lesions of the oropharyngeal mucosa. The patient described right eye irritation, pain with blinking, redness, and watery discharge. He denied headache, photophobia, decreased vision, and other visual symptoms. On the day of presentation, his mother noticed 2 ulcer-like lesions that had developed on the palpebral conjunctiva of the right lower eyelid.

Past medical history included attention deficit hyperactivity disorder and generalized anxiety disorder. Medications included amoxicillin for a recent ear infection, fluoxetine, and methylphenidate. Past ocular history was unremarkable other than mild refractive error.

His systemic exam was remarkable only for a widespread papulovesicular rash consistent with chickenpox.

**FIGURE 1.**
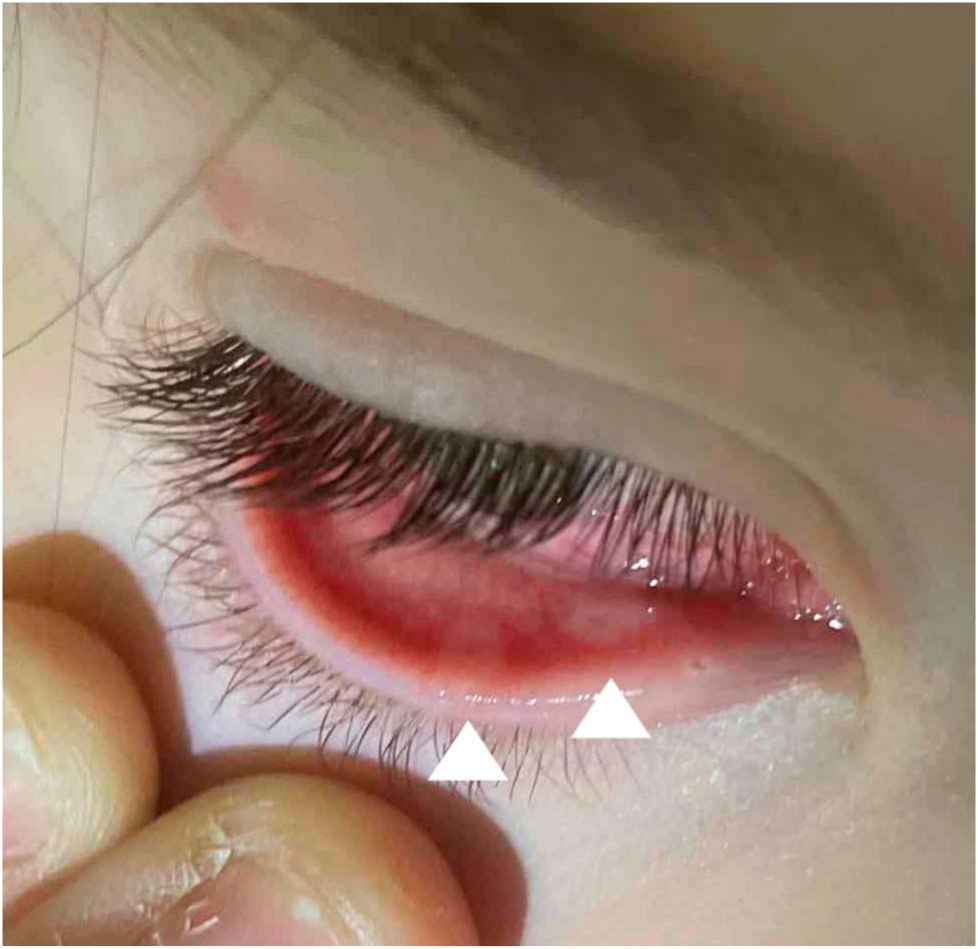
Right eye. Two discrete lesions of the inferior conjunctiva (arrowheads) were apparent at presentation. There was also a skin lesion on the lateral aspect of the upper eyelid.

**FIGURE 2.**
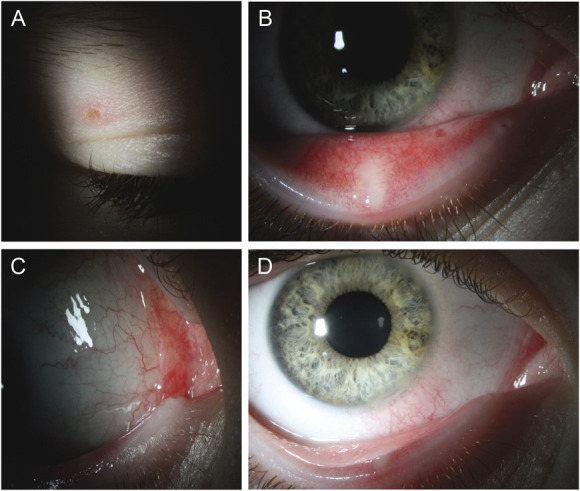
Clinical photographs 2 days after initial presentation. A, Right upper eyelid lesion. B, Two resolving lesions on the inferior palpebral conjunctiva. C, Injection and an indiscrete lesion of the plica semilunaris. D, External photograph showing the marginal inferior conjunctival lesion and sectoral injection of the inferonasal perilimbal bulbar conjunctiva, without a discrete visible perilimbal lesion.

Ophthalmologic exam revealed 20/20 uncorrected near vision bilaterally, symmetric and brisk pupillary responses without a relative afferent pupillary defect, and intraocular pressures of 19 and 17 mm Hg in the right and left eye, respectively. Extraocular movements and confrontation visual fields were normal. In the right eye, there were conjunctival follicles and 2 round, raised white lesions on the inferior palpebral conjunctiva measuring approximately 2 mm in diameter (Fig. 1). Additionally, there were 2 flat lesions on the bulbar conjunctiva that under light microscopy appeared as focal conjunctival injection: 1 adjacent to the limbus at 5 o'clock, and at the plica semilunaris. All of these lesions stained avidly with fluorescein. The mild injection of the limbal and nasal canthal lesions blanched with topical phenylephrine. There were no corneal defects and the eye was otherwise quiet without evidence of intraocular involvement. There were scattered vesicles across the patient's face that involved the external eyelids of both eyes (Fig. 2A). Swabs of the lesions on the palpebral conjunctiva were obtained and tested for herpes simplex virus, VZV, and orthopox virus using polymerase chain reaction (PCR), as well as bacterial gram stain and culture and nucleic acid amplification testing for gonorrhea and chlamydia. The patient was started on treatment with erythromycin ointment 3 times per day in the symptomatic right eye.

At a follow up visit 3 days later, he reported significant improvement in his right eye pain. Tests of visual function remained excellent and symmetric in both eyes. The lesions near the limbus and plica semilunaris had diminished, while the conjunctival lesions remained but had reduced in size (Figs. 2B and D). VZV PCR of the conjunctival swab returned positive, while the other viral and bacterial studies were negative. He continued erythromycin ointment for 4 more days, and 1 week later, the patient's ocular symptoms and rash had fully resolved.

## DISCUSSION

This case highlights ocular surface morbidity associated with primary VZV infection, a once-ubiquitous disease in the United States that is becoming increasingly rare due to the widespread vaccination of children using a live virus vaccine starting at 12 months of age.^1^ Ocular involvement beyond the eyelid is uncommon in chickenpox. Jordan, et al^5^ described 24 children examined in a pediatric ophthalmology practice who presented with ocular manifestations of primary varicella infection. Of these patients, 15 had vesicular lesions of the external eyelids, 8 had conjunctival involvement, 2 had corneal involvement, and 6 exhibited anterior uveitis. Of the 8 with conjunctival involvement, 75% had a single raised pock-like lesion at the limbus while 25% had a non-elevated limbal lesion similar to that described in our case.^5^ However, none of the patients described by Jordan, et al were reported to have multiple conjunctival lesions like this patient. The conjunctival lesions in this patient were thought to most likely be ruptured vesicles.

Given the infrequency of ocular surface involvement in chickenpox, there are no clear guidelines for its management. Ocular surface lesions have been reported to resolve without treatment,^5^ however, given the patient's discomfort and compromise of the conjunctival epithelium, lubrication with antibiotic ointment was initiated to reduce the likelihood of secondary infectious conjunctivitis and cicatricial sequelae. Topical antiviral treatment (eg, ganciclovir) can be considered in more severe cases.

The differential diagnosis for our patient's case included other causes of viral conjunctivitis that can present with similar skin lesions, including herpes simplex virus infection, monkeypox, and molluscum contagiosum. It was also prudent to rule out bacterial conjunctivitis, particularly chlamydial conjunctivitis, given the predominance of follicles. While chlamydial conjunctivitis typically presents less acutely, it is important to consider in the differential as a potential indicator of abuse. PCR of a conjunctival swab is the diagnostic modality of choice to differentiate these infections. In this case, there was high pre-test probability of VZV conjunctivitis given that this patient's history and cutaneous eruption were typical of chickenpox, and a positive VZV PCR result confirmed the diagnosis. However, the classic presentation of primary varicella zoster infection is becoming less frequent; varicella infection in immunized individuals, known as breakthrough varicella, can present without classic chickenpox features, including fewer skin lesions that are more maculopapular than vesicular.^1^

Systemic complications of varicella include bacterial superinfection of superficial lesions, neurologic complications, pneumonia, hepatitis, glomerulonephritis, and even death. Age (both infancy and adulthood), immunocompromise, and pregnancy are risk factors for developing severe or fatal disease^1^; these at-risk individuals rely heavily on herd immunity since live attenuated vaccines are contraindicated in certain conditions. Our patient had no signs of or risk factors for severe disease, so systemic antiviral treatment was not indicated. Though uncommon, even breakthrough varicella infection can cause severe systemic disease (especially among high-risk populations), which highlights the importance of accurate diagnosis.^1^
